# Skin single-cell transcriptomics reveals a core of sebaceous gland-relevant genes shared by mice and humans

**DOI:** 10.1186/s12864-024-10008-8

**Published:** 2024-02-03

**Authors:** Torsten Thalheim, Marlon R. Schneider

**Affiliations:** 1 Interdisciplinary Institute for Bioinformatics (IZBI), University of Leipzig, Härtelstr. 16-18, 04107 Leipzig, Germany; 2 Present Address: Deutsches Biomasseforschungszentrum gGmbH, Torgauer Str. 116, 04347 Leipzig, Germany; 3 Institute of Veterinary Physiology, University of Leipzig, An den Tierkliniken 7, Leipzig, 04103 Germany

**Keywords:** Sebaceous gland, Skin, Single-cell transcriptomics, Bioinformatics

## Abstract

**Background:**

Single-cell RNA sequencing (scRNA-seq) has been widely applied to dissect cellular heterogeneity in normal and diseased skin. Sebaceous glands, essential skin components with established functions in maintaining skin integrity and emerging roles in systemic energy metabolism, have been largely neglected in scRNA-seq studies.

**Methods:**

Departing from mouse and human skin scRNA-seq datasets, we identified gene sets expressed especially in sebaceous glands with the open-source R-package oposSOM.

**Results:**

The identified gene sets included sebaceous gland-typical genes as *Scd3*, *Mgst1*, *Cidea*, *Awat2* and *KRT7*. Surprisingly, however, there was not a single overlap among the 100 highest, exclusively in sebaceous glands expressed transcripts in mouse and human samples. Notably, both species share a common core of only 25 transcripts, including mitochondrial and peroxisomal genes involved in fatty acid, amino acid, and glucose processing, thus highlighting the intense metabolic rate of this gland.

**Conclusions:**

This study highlights intrinsic differences in sebaceous lipid synthesis between mice and humans, and indicates an important role for peroxisomal processes in this context. Our data also provides attractive starting points for experimentally addressing novel candidates regulating sebaceous gland homeostasis.

**Supplementary Information:**

The online version contains supplementary material available at 10.1186/s12864-024-10008-8.

## Introduction

In light of its complex biology and its involvement in numerous diseases, the mammalian skin provides an attractive paradigm for research in a variety of topics, including stem cell biology, cell adhesion and inflammation [1, 2]. The skin fulfills numerous key functions, which include the formation of a physical, chemical and biological protective interface with the external environment, supporting body thermoregulation by harboring hairs and secreting sweat and lipids, and by acting as a sensory organ. Structurally, it consists of two layers separated by a basal membrane [2, 3]. The upper epidermis is a multilayered stratified epithelium, constantly renewed by a balance between self-renewal of basal cells and their differentiation, which includes detachment from the basal membrane and terminal differentiation in a process termed cornification. The lower dermis consists essentially of fibroblasts in an extracellular matrix and harbors structures such as hair follicles with associated sebaceous glands (SG), sweat glands, nerves, and sensory receptors as wells as blood and lymphatic vessels. The dermis also includes a variety of immune cells as dendritic cells, macrophages, T-cells, mast cells, and eosinophils. The lowest part of the dermis is continuous to a fat layer, called dermal white adipose. The skin additionally includes different types of stem cells, muscle cells (responsible for the goosebumps) and pigment-containing melanocytes; altogether, the skin is composed by more than 50 different cell types [4].

These features make the skin a particularly attractive study object for methods as single-cell RNA sequencing (scRNA-seq), which can deal with the heterogeneity of RNA transcripts and assess different cell types and their functions in highly organized tissues [5]. scRNA-seq has been extensively applied to assess different aspects of human skin pathophysiology, such as the characterization of epidermal stem cells and the study of wound healing, fibrotic diseases or skin cancer [6–8]. A number of studies also assessed single cell transcripts of mouse skin under a variety of conditions, providing novel insights into epidermal homeostasis, hair follicle cycling, or wound healing [4, 9–11].

SG are exocrine glands associated with hair follicles, whose secretion (sebum) lubricate and protect the skin and hair, in addition of having other (rather uncharacterized) antimicrobial and antioxidative protective functions [12]. SG cells (sebocytes) can be divided in distinct populations according to their maturation stage: the peripheral zone is composed of flat, mitotically active cells in contact with the basal lamina. Under the influence of hormones and local factors, peripheral cells start synthesizing and accumulating lipids in the form of intracellular droplets and are displaced towards the middle of the gland, forming the maturation zone. As lipid accumulation proceeds, cells undergo an extraordinary increase in volume, become distorted in shape, display pyknotic nuclei, and organelles become generally unrecognizable. This process culminates in membrane disruption and release of the cellular contents at the center of the gland, in a process called holocrine secretion [13–15]. The cell debris reach the skin surface via the sebaceous duct and the hair follicle canal; during this journey, the oily product of sebocyte disruption may pick up additional substances and its lipids may by modified, resulting in the formation of sebum. Sebum composition differs significantly from that of adipocyte-produced fat, and is composed by large amounts of triglycerides, diglycerides and free fatty acids (57%), wax esters (26%), squalene (12%), and cholesterol (2%) [16]. Wax esters and squalene are typical for sebum and are normally not found elsewhere in the body. Interestingly, sebum fatty acids show unusual saturation and branching patterns [17, 18]. As the proportion of the different lipid classes significantly differs among species [19], comparisons between human and laboratory animal SG and sebum is challenging.

In the last years, the key role of SG in modulating skin immunology and inflammatory responses [20, 21] or its neuroendocrine regulation [22] became increasingly appreciated. While interest in SG increased substantially, the gland and its product are considerably less characterized than other skin compartments as the hair follicle, and commonly overlooked when studying skin biology. For instance, it was shown that epidermal stem cell differentiation is modulated by specific lipid species [23], but this study was restricted to epidermal lipids and did not include sebaceous lipids, which is regrettably considering that the latter make out the majority of lipids at the skin surface. The latter circumstance was the rationale for the recent study by Inoue and colleagues, who assessed transcripts in skin surface lipids and found predominantly mRNAs derived from SGs [24]. In another study, laser-capture microdissection followed by bulk RNAseq (LCM-RNAseq) was used to define the SG gene transcriptional profiles [25].

Except for a recent study focusing on PPARG-positive mouse sebocytes [26], SG transcripts have not been at the focus of scRNA-seq studies so far. While in some cases sebocytes were absent in the sample analyzed because of the chosen skin location [27, 28], other scRNA-seq studies probably did capture sebocytes transcripts, but these were either not annotated or not followed in detail because the study had a widely different focus [10, 11, 29]. Here, we re-assessed two well-annotated scRNA-seq data sets from human [30] and mouse [9] skin to assess and compare SG transcripts in these species. We selected these studies because they provide a comprehensive annotation of epidermal samples that allows to identify individual cells comprising the SG.

## Methods

In this study we analysed already existing scRNA-seq data of human [30] and mouse [9] skin from a sebaceous gland perspective. Although the original works were not focussed on SG, the authors identified such samples in their approach. According to the authors, all experiments were carried out in accordance to local ethical and legal regulations. All sample handling and sequencing experiments belong to the original work; we therefore refer to these publications for details about the experimental setup.

We applied the statistical software R [31] and its packages biomaRt [32] and oposSOM [33] to re-analyze both datasets to unravel the characteristics of SG transcripts in comparison to the other labelled epidermal regions. In addition we used the R package ggplot2 [34] to visualize our results. Furthermore, the identification of homologue genes is part of biomaRt [32], whereas the oposSOM-framework [33] was used to explore the gene expression in both datasets individually. oposSOM uses various metrics, such as logFC or min–max, for sample ranking and geneset analysis and has been applied successfully in the past to a variety of gene expression analysis such as cancer specific modifications [35, 36] and in particular also to characterize scRNA-seq samples [37, 38].

The mouse scRNA-seq data [9] consisted of epidermal cells from dorsal skin of C57BL/6 wild-type mice during second telogen at around 8 weeks of age. This dataset consists of 13 epidermal regions comprising approximately 1400 cells and 26000 genes. The human epidermal scRNA-seq samples [30] included normal surgical tissue discards from circumcisions, reduction abdominoplasties and mammoplasties, and scalp excisions. The human dataset consists of 7 epidermal regions comprising approximately 93000 cells and 19000 genes.

All scRNA-seq samples are provided as total reads per cell. The base-10 logarithmic total reads were analyzed by the oposSOM R-package [33]. In a first attempt this data is normalized and genes with constant expression are removed from the final dataset. Thereby, the genes are clustered into metagene profiles with each metagene serving as a representative of a cluster of similarly expressed genes. For both datasets, we used a grid of 30 × 30 metagenes and applied most parameters with default settings provided in the oposSOM-framework. Deviating from the default options, we specified sample groups according to the subpopulations denoted in the original work [9, 30], increased the parameter training.extension and fixed the parameter dim.1stLvlSom (see Supplementary Table 1). Thus, oposSOM provides various categories of interesting areas of metagenes ranked by internal criteria, such as logFC differences or the GSZ score.

Finally, in each dataset we analysed unsupervised the mouse and human SG-samples only. Unsupervised implies that we did not predefine sample groups. With this setting, oposSOM sorts the samples by internal similarity criteria and assign them afterwards to a sufficient number of distinct groups. To avoid a bad separation following from bias through local similarities or too strict segregation, we increased the parameter training.extension (see Supplementary Table 1).

We used the R-package ggplot2 [34] to visualize the violin expression patterns. There we applied scale = ”width” to overcome the different amount of samples (two datasets were split into four subsets: SG cells and others for mouse and human data) and normalized any violin to the same maximum width.

For demonstrating the localization of selected proteins by immunohistochemistry, we assessed the Human Protein Atlas (https://www.proteinatlas.org/). Details of this open access resource are provided elsewhere [39].

## Results

### Sebaceous gland cells are heavily underrepresented in skin scRNA-seq data

**Fig. 1:**
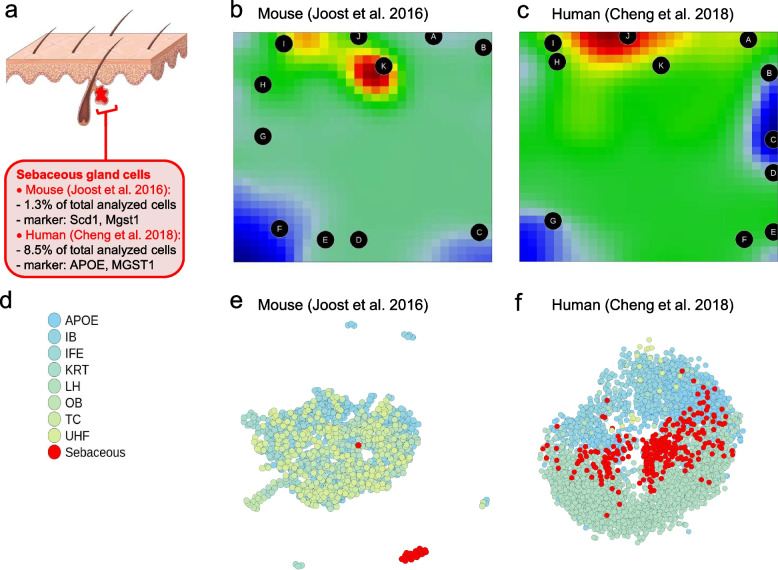
Identification of mouse and human sebaceous gland cells with oposSOM. **a** Scheme of the main skin epithelial compartments analysed by scRNA-seq, the defining marker and the percentage of identified sebaceous gland cells relative to all cells analysed. The skin outline was drawn by using a picture from Servier Medical Art (licensed under a Creative Commons Attribution 3.0 Unported License; https://creativecommons.org/licenses/by/3.0/). **b** Expression portraits of the sebaceous gland populations in mouse and **c** human. **d** A combined legend of cell populations from mouse [9] and human [30]. Tissues that are split in several subgroups, such as uHF-1, uHF-2, uHF-3, are aggregated to a common group. Sebaceous gland cells are represented in red**.** APOE: Apolipoprotein E (human samples only); IB: Inner bulge (human and mouse samples); IFE: Interfollicular epidermis (human and mouse samples); KRT: Keratin 15/glutathione peroxidase 4 (human samples only); LH: Langerhans (mouse samples only); OB: Outer bulge (human and mouse samples); TC: T-cells (mouse samples only); UHF: Upper hair follicle (human and mouse samples). Correlation trees demonstrate mouse (**e**) and human (**f**) sebaceous gland cell clustering and segregation from the remaining population

The assessed mouse scRNA-seq data [9] encompassed interfollicular and follicular epidermal cells from dorsal skin samples. Unsupervised clustering resulted in thirteen main cell populations, including different compartments of the epidermis and hair follicle, immune cells, and SG cells. SG cells were characterized by *Scd1*/*Mgst1* expression and made up only 1.3% (19 / 1422) of total analysed cells (Fig. 1a). The human epidermal scRNA-seq samples [30] originated from adult scalp skin, adult truncal skin, and neonatal foreskin. Scalp samples were further categorized into seven subpopulations inspired by the clustering provided in the mouse scRNA-seq dataset [9], of which the follicular cluster of the scalp samples turned out to include SG cells [30]. Resembling the mouse study, human SG cells were characterized by *APOE*/*MGST1* expression and made up only 8.5% (260 / 3044) of total analysed cells (Fig. 1a). Both mouse and human SG transcripts, as originally analysed, build up a discrete population when visualized with t-distributed stochastic neighbour embedding (Supplementary Fig. 1). We therefore took advantage of the available scRNA-seq data and the specified SG subpopulations, and analysed the whole data set with the machine learning open-source R-package oposSOM [33].

### oposSOM-based scRNA-seq data analysis distinguishes SG cells

oposSOM characterizes each subpopulation, denoted as group, by an individual expression portrait, each of them providing areas with strongly (red) expressed metagenes. The expression portraits of the SG population in mouse (Fig. 1b, Supplementary Fig. 2) and human (Fig. 1c, Supplementary Fig. 3) scRNA-seq samples are thereby clearly discriminated from all remaining cell populations. The SG cell clustering and segregation is also illustrated by corresponding correlation trees (Fig. 1d,e,f). Furthermore, for the entire dataset, oposSOM identifies regions of metagenes, denoted as spots, that are expressed strongly in a subset of samples. In contrast to the pre-defined groups, these spots may compose samples from various populations.

### No single overlap in the 15 highest expressed genes in mouse and human sebaceous glands

**Fig. 2:**
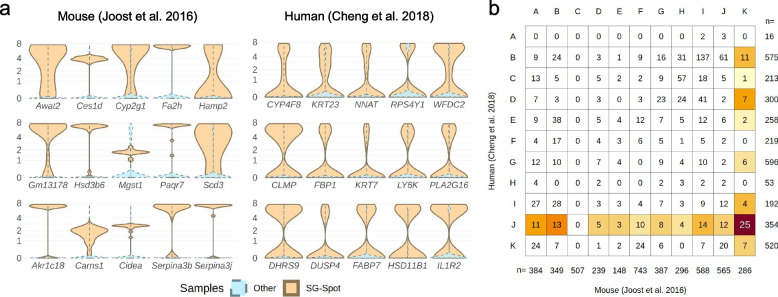
Identification of sebaceous gland spots and the highest expressed genes. **a** The top 15 enriched genes in mouse [9] and human [30] sebaceous gland exclusive spots. Samples assigned to the sebaceous gland spot show a different expression pattern than other samples. **b** In both mouse and human datasets, oposSOM identified one spot that consists of sebaceous glands cells almost exclusively, the color code indicates (red: maximal) the amount of homologous genes in the corresponding spot of the other species

To assess which genes are commonly expressed at high levels in mouse and human SG exclusively, we selected the 15 transcripts with the highest oposSOM rank in both spots (Fig. 2a) consisting primarily of SG cells. As expected, the mouse dataset included a number of genes previously known to be expressed specifically or at high levels in SG (*Scd3, Mgst1, Cidea, Awat2*), and the human dataset included the human SG-specific marker *KRT7* as well as *FABP7*, a protein involved in fatty acid uptake, transport, and metabolism. Several other SG-typical transcripts were also clearly enriched in the human SG dataset compared to the remaining cells, including *ELOVL5* (logFC 1.89), *PLIN2* (logFC 1.08), *MGST1* (logFC 2.54), and *CIDEA* (logFC 1.21). However, to our surprise, there was not a single overlap in the listed genes when comparing both species. In fact, the first overlap is represented by *CLMP*, which is ranked at position 12 in the human samples and at position 111 in the mouse samples.

### Twenty-five commonly expressed genes characterize mouse and human sebaceous glands

Adhering to our goal to identify common genes in mouse and human SG, we next compared both spots for similar gene ontology sets (via oposSOM ranking) and genes via homology mapping provided with the R-package biomaRt [32]. This processing identified 25 mouse and human homologue genes (Fig. 2b), which are depicted with their respective FC-values and main attributes in Table 1 and Extended Table 1 (Additional file). The identified transcripts include genes whose expression in SG seems logical considering their high lipid metabolism, such as *ELOVL7* and *LPCAT3*. However, for the most part, the identified genes have not been associated with this gland so far (Table 1).

**Table 1:** Transcripts jointly upregulated in human and mouse sebaceous gland cells. An extended table in machine readable format is provided in the supplement (Extended Table 1)

Gene symbol	logFC mouse	logFC human	Important protein features and functions	Key reference
ACYP2	2.563261	0.253610	Acylphosphatase with potential roles in Ca^2+^ homeostasis, pyruvate metabolism, and apoptosis	[52]
APMAP	5.839221	2.652425	Membrane protein highly up-regulated during adipogenic differentiation	[53]
CLMP	3.983276	2.385026	Tight junction protein up-regulated during adipogenic differentiation	[54]
CLSTN3	2.457828	0.783045	Adhesion protein of the postsynaptic membrane, expressed at high levels in human adipose tissue	[55]
ELOVL5	3.473510	1.559639	Catalyzes the initial, rate-controlling reaction for the elongation of polyunsaturated long-chain fatty acids	[56]
FKBP5	4.750235	0.571134	Co-chaperone for androgen and corticosteroid receptors, involved in steroid signaling	[57]
HIBADH	4.917526	0.998703	Enzyme involved in the oxidation of the branched-chain amino acid valine	[58]
JAGN1	2.641510	0.405730	Endoplasmic reticulum-resident transmembrane protein essential for neutrophil function	[59]
LPCAT3	5.854686	0.686207	Enzyme involved in phospholipid remodeling and control of phospholipid fatty acid composition	[60]
MCCC1	3.849271	0.458193	Catalyzes a critical step for leucine and isovaleric acid catabolism	[61]
MCCC2	2.836710	0.564344	Catalyzes a critical step for leucine and isovaleric acid catabolism	[62]
MOSPD1	4.716018	1.156748	Transmembrane protein with roles in the proliferation of mesenchymal cells	[63]
PEX11A	2.931246	0.644996	Involved in protein import into peroxisomes, as well as peroxisome biogenesis and proliferation	[64]
PEX16	5.410875	1.139134	Involved in protein import into peroxisomes, as well as peroxisome biogenesis and proliferation	[65]
PMPCB	1.826678	0.849944	Main peptidase responsible for cleaving the mitochondrial import signal from numerous proteins	[66]
PYGB	4.442553	0.489594	Catalyzes the rate-limiting step of glycogenolysis by releasing glucose-1-phosphate	[45]
RBM47	4.558278	1.495389	RNA-binding protein, regulates posttranscriptional gene expression in a variety of biological processes	[67]
RNF5	2.108283	0.277768	Membrane-bound E3 ubiquitin-protein ligase that mediates ubiquitination of target proteins	[68]
SAMM50	3.730143	2.190231	Mitochondrial outer membrane protein required for correct cristae structure and proper assembly of the respiratory chain complexes	[69]
SERPINB1	2.016565	0.823378	Intracellular protein that protects the cell from proteases released into the cytoplasm	[70]
SLC1A3	1.969376	0.613999	Transporter that mediates the uptake of acidic amino acids (Asp/Glu)	[71]
SND1	1.892262	0.615016	Transcriptional coactivator present in multiple organelles, involved in lipid metabolism	[72]
TMED4	1.606262	0.685340	Endoplasmic reticulum protein involved in heat-shock and oxidative stress responses	[73]
TMEM97	4.939064	1.504585	Endoplasmic reticulum-resident transmembrane protein involved in cholesterol homeostasis	[74]
TRUB2	1.534258	0.464128	Mitochondrial pseudouridine synthase, essential for post-transcriptional RNA modification	[75]

**Fig. 3:**
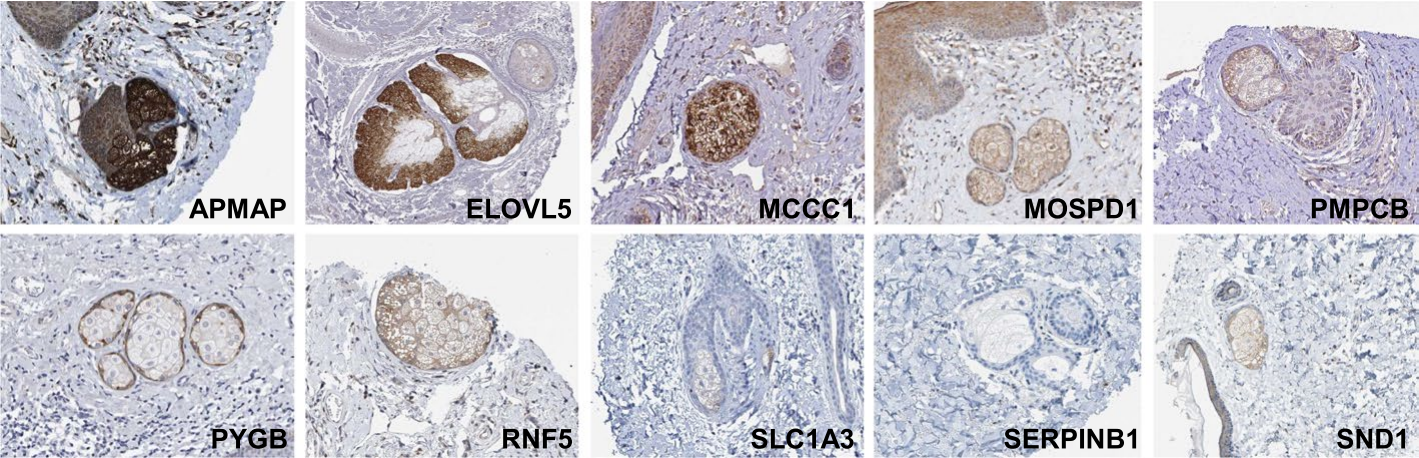
Expression of the protein encoded by the mouse/human core genes in human sebaceous glands by immunohistochemistry. Skin samples from the Human Protein Atlas (proteinatlas.org) immunohistochemically stained for the corresponding protein were screened for the presence of sebaceous glands

To confirm the expression of the corresponding proteins, we accessed the Human Protein Atlas [39]. While SGs are not annotated in this database, several immunohistochemically-stained skin samples include SG, thus allowing an evaluation of protein expression of the identified transcripts. In total, 10 out of the 25 candidate proteins could be evaluated, and, except for SERPINB1, expression in SG cells could be confirmed for all proteins (Fig. 3). Adipocyte plasma membrane–associated protein (APMAP) and methylcrotonoyl-CoA carboxylase subunit alpha, mitochondrial (MCCC1) are particularly strongly expressed in the whole SG, while elongation of very long chain fatty acids protein 5 (ELOVL5) shows intensive staining restricted to the peripheral layers of the SG. Weaker, but homogeneous expression was observed for motile sperm domain-containing protein 1 (MOSPD1), mitochondrial-processing peptidase subunit beta (PMPCB), ring finger protein 5 (RNF5) and staphylococcal nuclease and tudor domain containing 1 (SND1), while glycogen phosphorylase B (PYGB) expression was clearly restricted to the utmost peripheral layer of the gland (Fig. 3).

### Peroxisomal genes are strongly enriched in mouse and human sebaceous gland cells

Based on the ranking provided by oposSOM, we identified within the TOP10 ranked Gene Ontology (GO) sets, four GO sets (Table 2) associated with the SG cells in both datasets. It was again not surprising to see fatty acid metabolism and mitochondrial processes among the enriched GOs. Peroxisomal processes, in contrast, have not been so far considered as a hallmark of SG activity. Undoubtedly, this general observation of peroxisomal activity in all SG cells corresponds to an enrichment of peroxisomal genes inside the human (22/124, *p* = 2e-18) and the mouse (8/114, *p* = 0.005) spots consisting almost exclusively of SG cells. The TOP30 representatives of the GO-set peroxisome identified in all SG samples are listed separately for mouse and human genes in Table 3 (see the Additional file Extended Table 3 for fold-change values and gene IDs). TOP30 genes allocated in the SG spots too are underlined.

**Table 2:** Gene ontology sets associated with mouse and human SG samples

GO Term	GO ID
fatty acid metabolic process	GO:0006631
mitochondrial inner membrane	GO:0005743
mitochondrion	GO:0005739
peroxisome	GO:0005777

**Table 3:** Peroxisomal transcripts (GO:0005777) upregulated in human and mouse sebaceous gland cells. Underlined transcripts are allocated in the SG spots too. An extended table in machine readable format is provided in the supplement (Extended Table 3)

Human [30]		Mouse [9]	
**Gene symbol**	**logFC**	**Gene symbol**	**logFC**
*IDH1*	1.714958	*Crat*	6.019170
*TKT*	1.708468	*Tmem135*	5.443929
*CROT*	1.153379	*Pex16*	5.410875
*PEX16*	1.139134	*Pxmp4*	5.357285
*GSTK1*	1.100090	*Hsd17b4*	5.231357
*ECH1*	1.088596	*Eci2*	5.167680
*ACAA1*	0.927157	*Acoxl*	5.061069
*ISOC1*	0.913967	*Mvd*	5.009957
*ACSL4*	0.894847	*Far2*	4.740132
*HSD17B4*	0.866348	*Tkt*	4.727190
*HSDL2*	0.791853	*Sod1*	4.685854
*CAT*	0.774506	*Idi1*	4.627176
*PEX11A*	0.644996	*Fdps*	4.543885
*PMVK*	0.641051	*Acaa1b*	4.528534
*IDH2*	0.575469	*Acox1*	4.436426
*HACL1*	0.542409	*Cat*	4.320156
*PXMP2*	0.512519	*Abcd3*	4.290786
*DHRS4*	0.508109	*Acsl4*	4.278983
*ACSL3*	0.504865	*Nudt17*	4.227694
*ECI2*	0.472204	*Ech1*	4.190630
*DHRS7B*	0.447013	*Idh1*	4.087035
*ACSL1*	0.440021	*Hsdl2*	4.059215
*MFF*	0.429729	*Acox3*	3.962428
*AGPS*	0.410743	*Pex3*	3.925956
*NUDT12*	0.402126	*Pex19*	3.918628
*VWA8*	0.394270	*Phyh*	3.868502
*PEX19*	0.389455	*Pex11g*	3.840121
*PEX7*	0.388951	*Decr2*	3.817467
*ACOX1*	0.381587	*Pmvk*	3.803012
*SCP2*	0.365516	*Pnpla8*	3.658509

**Fig. 4:**
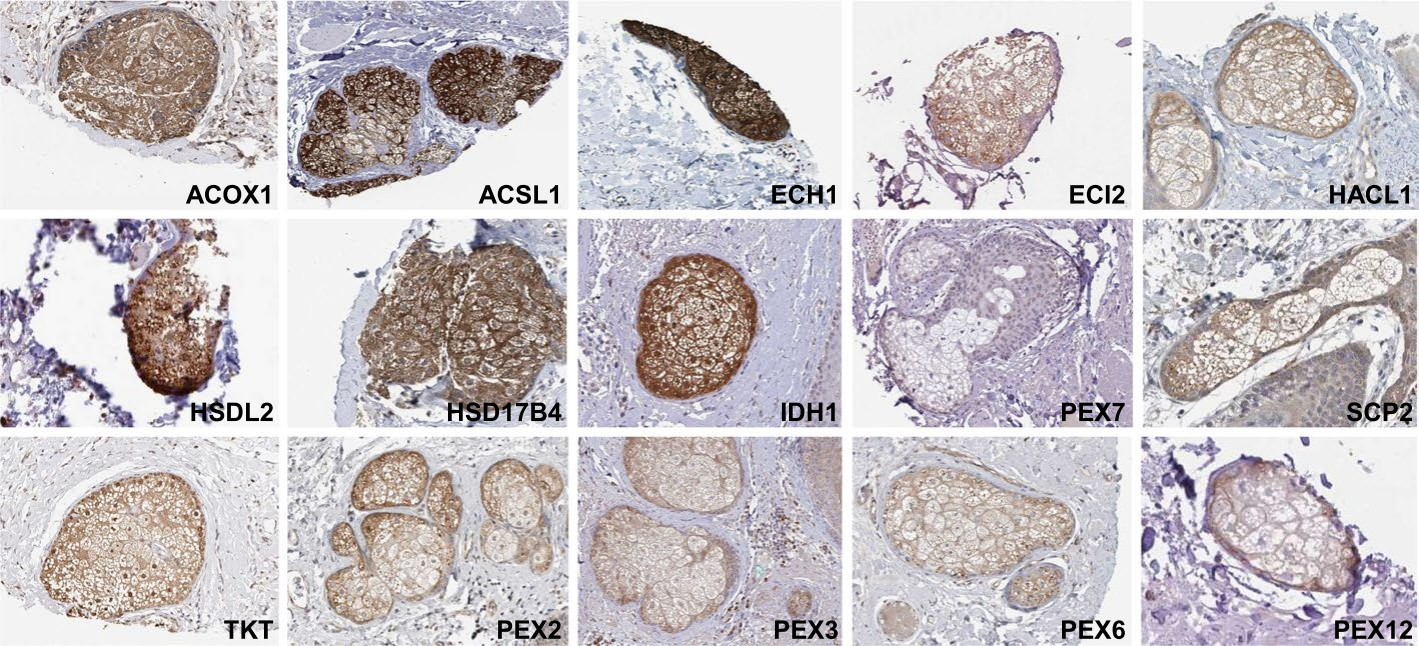
Expression of proteins encoded by the genes of the GO term “Peroxisome” in human sebaceous glands by immunohistochemistry. Skin samples from the Human Protein Atlas (proteinatlas.org) immunohistochemically stained for the corresponding protein were screened for the presence of sebaceous glands

To confirm protein expression of the identified genes in human SG in the skin in situ, we again assessed the Human Protein Atlas [39]. Due to their presence in selected immunohistochemical-stained skin samples, it was possible to evaluate staining in SG for 11 out of the 30 peroxisomal genes assessed, and all of the assessable proteins except for peroxisomal targeting signal 2 receptor, also known as peroxin 7 (PEX7), revealed to be expressed with a range of staining intensities (Fig. 4). Although staining for PEX7 was negative, we were able to confirm expression of other peroxins (PEX2, PEX3, PEX6, and PEX12), thus confirming the presence of numerous peroxisomal proteins in human SG (Fig. 4).

### No additional insights by sebaceous gland-specific sample exploration

In a final analysis, both SG populations only were explored in an unsupervised manner. In this approach, oposSOM classifies the cells according to internal criteria. In this process the human samples are allocated in six groups and seven spots, while the mouse samples are characterized by four groups and nine spots. Comparing their overlap, it seems that the unsupervised composed groups do not allow a more detailed functional insight into the SG transcriptome (Supplementary Fig. 4). Considering the small number (*n* = 19) of SG samples in the mouse dataset, this statement might be revised once more numerous samples become available.

## Discussion

In this study, we harnessed skin scRNA-seq data to throw light on the transcriptional landscape of mouse and human SG cells. The employed mouse scRNA-seq dataset [9], while arguably rather small for more advanced bioinformatic analyses (it includes only 19 individual SG cell transcripts), has been shown to be highly predictive for the identification of sebocyte-specific proteins [40]. Furthermore, the genes with the highest expression in our analysis encode numerous proteins of recognized relevance in SG physiology, such as Scd3, Mgst1, Cidea, and Awat2. By using the machine learning open-source R-package oposSOM [33], we were able to derive SG-specific cell characteristics from both mouse [9] and human [30] datasets. Hereby, oposSOM takes advantage of these well-annotated datasets and characterizes each group, denoted as tissue subpopulation by the authors, through an individual expression portrait, each of them providing areas with strongly expressed metagenes. Conversely, these areas of strongly expressed metagenes are available for the entire dataset too, and will be used to identify cell subsets with similar expression pattern which are incorporated to individual spots in this process. In that way, these spots comprise cells that preferentially express a certain gene set. Yet, a spot may either reflect an originally defined tissue-allocated cell population, or it may consist of cells from various tissues. In this work we focused on those spots that comprise almost exclusively cells allocated in the SG. Notably, these spots do not include all SG cells. Moreover, both datasets contain spots comprising cells from various tissues, including SG cells.

The 15 genes with the highest expression in the mouse spot associated mainly with SG samples encode a range of proteins intimately associated with SG, such as Scd3 [12], Mgst1 [41], Cidea [42], and *Awat2* [43], while the human dataset included only KRT7 [44] as a prominent human SG-specific marker. Nevertheless, numerous other SG-typical transcripts were massively enriched in the human SG dataset (*ELOVL5, PLIN2, MGST1*, and *CIDEA*, among others). Whether the difference in the density of SG-typical transcripts in the TOP15 list reflects an unfavorable choice of markers in the human samples, resulting in the inclusion of non-sebaceous cells in the subset, remains to be determined. Anyway, quite astonishingly, there was not a single overlap when both species were compared, indicating that our analysis revealed a previously unknown core of genes relevant for both mouse and human SG. These include numerous mitochondrial and peroxisomal genes involved in fatty acid, amino acid, and glucose processing, thus highlighting the high metabolic rate of this gland. While SGs are not annotated in the Human Protein Atlas [39], database, we were able to assess the protein expression of part of the identified genes by screening immunohistochemically-stained skin samples for the presence of SG. Altogether, 10 out of the 25 candidate proteins could be evaluated, and, except for SERPINB1, expression in SG cells could be confirmed at different intensities and in part with a conspicuous expression pattern for the remaining proteins.

As an example, expression of PYGB, which catalyzes the rate-limiting step of glycogenolysis [45], was clearly restricted to the uttermost peripheral layer of the glands. Glycogen presence in SG has been described decades ago [46], and it is known that this glucose polymer is an important substrate for sebum synthesis [47]. Our finding highlights a potential role for glycogen as a substrate for sebaceous energy production and biosynthesis, possibly in a way similar to that recently reported for hair follicles [48]. The identification of SLC1A3, a sodium-dependent, high-affinity amino acid transporter provides another clue for deciphering energy metabolism in SG, as previous studies revealed SLC1A3 to be involved in stem/progenitor cell activation in different skin niches, including the SG [49]. Thus, our systematic analysis of scRNA-seq data complements targeted studies, such as the recent identification of embigin as a modulator of SG cell adhesion and metabolism [40] departing form the data in Joost et al. [9], and provides novel starting points for experimentally addressing different aspects of SG homeostasis.

In contrast to the small overlap of transcripts within the SG associated spots, the ranking provided by oposSOM identified an overlap of four GO sets already within the Top 10 GO sets associated with the entire SG population in both datasets. While fatty acid metabolism and mitochondrial processes as enriched GOs were clearly expectable, it was rather surprising to also detect peroxisomal processes, as the latter have not been so far considered a hallmark of SG activity. Yet, peroxisomal genes were clearly enriched in both mouse and human SG genes with high expression, and expression of numerous peroxisome-relevant in human SG was confirmed by assessing immunohistochemical-stained skin samples from the Human Protein Atlas [39]. Peroxisomes are organelles present in almost all eukaryotic cells with manifold metabolic functions, including α- and β-fatty acid oxidation, synthesis of bile acids and plasmalogens, and response to oxidative stresses by handling reactive oxygen species [50]. Peroxisomes have been described in SG cells, where they may form extensive tubular aggregates [51]. The latter authors speculated that the wide variety in peroxisomal complex morphologies may in part explain the striking differences in sebum composition across species and across single individuals of the same species [18, 19]. Our data highlight the role of peroxisomes in SG metabolism and will stimulate the functional analysis of the identified proteins in sebaceous biology.

## Conclusions

We anticipate that future studies focusing on samples restricted to SG instead of whole skin will increase the resolution and the analytic power of the obtained data, thus providing novel insights into SG homeostasis, its secretory activity, and its interaction with other organs and tissues. In addition, our findings may also indicate novel potential targets for modulating SG activity in skin diseases involving altered SG function, as acne vulgaris, atopic dermatitis, and psoriasis.

### Supplementary Information

**Additional file 1: Supplementary Table 1.** Non-default oposSOM parameters applied in this work.

**Additional file 2:Supplementary Figure 1.** Original mouse (left) and human (right) epidermal cell transcriptome visualized with t-distributed stochastic neighbour embedding maps and corresponding legends showing sebaceous gland cells as discrete population. Figures are reproduced from Joost et al. (2016) and Cheng et al. (2018) under the CC BY-NC-ND license (http://creativecommons.org/licenses/by-nc-nd/4.0). **Supplementary Figure 2.** oposSOM-generated expression portraits (left) of the subpopulations in mouse scRNA-seq samples; sebaceous samples are thereby clearly discriminated from all remaining cell populations. Within the corresponding spots of strong expressed metagenes identified by oposSOM (right), Spot K represents the SG sample exclusive spot. **Supplementary Figure 3.** oposSOM-generated expression portraits (left) of the subpopulations in human scRNA-seq samples; sebaceous samples are thereby clearly discriminated from all remaining cell populations. Within the corresponding spots of strong expressed metagenes identified by oposSOM (right), Spot J represents the SG sample exclusive spot. **Supplementary Figure 4.** oposSOM-generated expression portraits of the sebaceous gland population in human (left) and mouse (bottom) scRNA-seq samples; The group overlap (top right) does not provide a distinct mouse to human group matching. The color code corresponds to the normalized appearance of homologous genes (red indicates the highest incidence).

**Additional file 3. **

**Additional file 4. **

## Data Availability

The mouse single-cell RNA-seq data [9] can be obtained in the Gene Expression Omnibus repository (GEO, https://www.ncbi.nlm.nih.gov/geo) under the accession number GSE67602. The human single-cell RNA-seq data [30] are available in the European Genome-Phenome Archive (EGA, https://ega-archive.org) under the accession number EGAS00001002927. The processed oposSOM data is available in the Leipzig Health Atlas (https://www.health-atlas.de) under the LHA ID 8HE9ACUKG1-3.

## References

[1] Watt FM (2014). Mammalian skin cell biology: at the interface between laboratory and clinic. Science.

[2] Hsu Y-C, Fuchs E (2022). Building and Maintaining the Skin. Cold Spring Harb Perspect Biol.

[3] Rognoni E, Watt FM (2018). Skin Cell Heterogeneity in Development, Wound Healing, and Cancer. Trends Cell Biol.

[4] Joost S, Annusver K, Jacob T, Sun X, Dalessandri T, Sivan U (2020). The Molecular Anatomy of Mouse Skin during Hair Growth and Rest. Cell Stem Cell.

[5] Jovic D, Liang X, Zeng H, Lin L, Xu F, Luo Y (2022). Single-cell RNA sequencing technologies and applications: A brief overview. Clin Transl Med.

[6] Theocharidis G, Tekkela S, Veves A, McGrath JA, Onoufriadis A (2022). Single-cell transcriptomics in human skin research: available technologies, technical considerations and disease applications. Exp Dermatol.

[7] Negri VA, Watt FM (2022). Understanding Human Epidermal Stem Cells at Single-Cell Resolution. J Invest Dermatol.

[8] Srivastava A, Bencomo T, Das I, Lee CS (2023). Unravelling the landscape of skin cancer through single-cell transcriptomics. Transl Oncol.

[9] Joost S, Zeisel A, Jacob T, Sun X, La Manno G, Lönnerberg P (2016). Single-Cell Transcriptomics Reveals that Differentiation and Spatial Signatures Shape Epidermal and Hair Follicle Heterogeneity. Cell Syst.

[10] Haensel D, Jin S, Sun P, Cinco R, Dragan M, Nguyen Q (2020). Defining Epidermal Basal Cell States during Skin Homeostasis and Wound Healing Using Single-Cell Transcriptomics. Cell Rep.

[11] Lin Z, Jin S, Chen J, Li Z, Lin Z, Tang L (2020). Murine interfollicular epidermal differentiation is gradualistic with GRHL3 controlling progression from stem to transition cell states. Nat Commun.

[12] Dahlhoff M, Camera E, Schafer M, Emrich D, Riethmacher D, Foster A (2016). Sebaceous lipids are essential for water repulsion, protection against UVB-induced apoptosis and ocular integrity in mice. Development.

[13] Schneider MR, Paus R (2010). Sebocytes, multifaceted epithelial cells: lipid production and holocrine secretion. Int J Biochem Cell Biol.

[14] Zouboulis CC, Picardo M, Ju Q, Kurokawa I, Torocsik D, Biro T, Schneider MR (2016). Beyond acne: Current aspects of sebaceous gland biology and function. Rev Endocr Metab Disord.

[15] Schneider MR (2016). Lipid droplets and associated proteins in sebocytes. Exp Cell Res.

[16] Smith KR, Thiboutot DM (2008). Thematic review series: skin lipids Sebaceous gland lipids friend or foe?. J Lipid Res.

[17] Stewart ME (1992). Sebaceous gland lipids. Semin Dermatol.

[18] Pappas A (2009). Epidermal surface lipids. Dermatoendocrinol.

[19] Nicolaides N (1974). Skin lipids: their biochemical uniqueness. Science.

[20] Zouboulis CC, Coenye T, He L, Kabashima K, Kobayashi T, Niemann C (2022). Sebaceous immunobiology - skin homeostasis, pathophysiology, coordination of innate immunity and inflammatory response and disease associations. Front Immunol.

[21] Güell M, Schneider MR (2023). In preprints: progress in sebaceous gland homeostasis, regeneration and immunomodulatory functions. Development.

[22] Clayton RW, Langan EA, Ansell DM, de Vos I, Gobel K, Schneider MR (2020). Neuroendocrinology and neurobiology of sebaceous glands. Biol Rev Camb Philos Soc.

[23] Vietri Rudan M, Mishra A, Klose C, Eggert US, Watt FM (2020). Human epidermal stem cell differentiation is modulated by specific lipid subspecies. Proc Natl Acad Sci U S A.

[24] Inoue T, Kuwano T, Uehara Y, Yano M, Oya N, Takada N (2022). Non-invasive human skin transcriptome analysis using mRNA in skin surface lipids. Commun Biol.

[25] Harris JC, Prouty SM, Nelson MA, Sung DC, Nelson AM, Seykora JT (2023). Laser Capture Microdissection-based RNAseq for Profiling Mouse and Human Sebaceous Gland Transcriptomes. J Invest Dermatol.

[26] Veniaminova NA, Jia Y, Hartigan AM, Huyge TJ, Tsai S-Y, Grachtchouk M, et al. Distinct mechanisms for sebaceous gland self-renewal and regeneration provide durability in response to injury. bioRxiv 2023. doi:10.1101/2023.05.05.539454. 10.1016/j.celrep.2023.113121 PMC10591672 37715952

[27] Han X, Wang R, Zhou Y, Fei L, Sun H, Lai S (2018). Mapping the Mouse Cell Atlas by Microwell-Seq. Cell.

[28] Wang S, Drummond ML, Guerrero-Juarez CF, Tarapore E, MacLean AL, Stabell AR (2020). Single cell transcriptomics of human epidermis identifies basal stem cell transition states. Nat Commun.

[29] Solé-Boldo L, Raddatz G, Schütz S, Mallm J-P, Rippe K, Lonsdorf AS (2020). Single-cell transcriptomes of the human skin reveal age-related loss of fibroblast priming. Commun Biol.

[30] Cheng JB, Sedgewick AJ, Finnegan AI, Harirchian P, Lee J, Kwon S (2018). Transcriptional Programming of Normal and Inflamed Human Epidermis at Single-Cell Resolution. Cell Rep.

[31] R Core Team. R: A Language and Environment for Statistical Computing. 2016. https://www.r-project.org/.

[32] Durinck S, Spellman PT, Birney E, Huber W (2009). Mapping identifiers for the integration of genomic datasets with the R/Bioconductor package biomaRt. Nat Protoc.

[33] Löffler-Wirth H, Kalcher M, Binder H (2015). oposSOM: R-package for high-dimensional portraying of genome-wide expression landscapes on bioconductor. Bioinformatics.

[34] Wickham H (2016). ggplot2: Elegant Graphics for Data Analysis.

[35] Klemm F, Maas RR, Bowman RL, Kornete M, Soukup K, Nassiri S (2020). Interrogation of the Microenvironmental Landscape in Brain Tumors Reveals Disease-Specific Alterations of Immune Cells. Cell.

[36] Herberg M, Siebert S, Quaas M, Thalheim T, Rother K, Hussong M (2019). Loss of Msh2 and a single-radiation hit induce common, genome-wide, and persistent epigenetic changes in the intestine. Clin Epigenetics.

[37] Ma K-Y, Schonnesen AA, Brock A, van den Berg C, Eckhardt SG, Liu Z, Jiang N (2019). Single-cell RNA sequencing of lung adenocarcinoma reveals heterogeneity of immune response-related genes. JCI Insight.

[38] Schmidt M, Mortensen LS, Loeffler-Wirth H, Kosnopfel C, Krohn K, Binder H, Kunz M (2021). Single-cell trajectories of melanoma cell resistance to targeted treatment. Cancer Biol Med.

[39] Uhlen M, Fagerberg L, Hallstrom BM, Lindskog C, Oksvold P, Mardinoglu A (2015). Proteomics Tissue-based map of the human proteome. Science.

[40] Sipilä K, Rognoni E, Jokinen J, Tewary M, Vietri Rudan M, Talvi S (2022). Embigin is a fibronectin receptor that affects sebaceous gland differentiation and metabolism. Dev Cell.

[41] Kobayashi T, Voisin B, Kim DY, Kennedy EA, Jo J-H, Shih H-Y (2019). Homeostatic Control of Sebaceous Glands by Innate Lymphoid Cells Regulates Commensal Bacteria Equilibrium. Cell.

[42] Schneider MR, Zhang S, Li P. Lipid droplets and associated proteins in the skin: Basic research and clinical perspectives. Arch Dermatol Res. 2016;308:1–6. 10.1007/s00403-015-1599-2 26437897

[43] Turkish AR, Henneberry AL, Cromley D, Padamsee M, Oelkers P, Bazzi H (2005). Identification of two novel human acyl-CoA wax alcohol acyltransferases: members of the diacylglycerol acyltransferase 2 (DGAT2) gene superfamily. J Biol Chem.

[44] Hinde E, Haslam IS, Schneider MR, Langan EA, Kloepper JE, Schramm C (2013). A practical guide for the study of human and murine sebaceous glands in situ. Exp Dermatol.

[45] Mathieu C, La Li de Sierra-Gallay I, Duval R, Xu X, Cocaign A, Léger T, et al Insights into Brain Glycogen Metabolism: THE STRUCTURE OF HUMAN BRAIN GLYCOGEN PHOSPHORYLASE 2016;291:18072–83. doi:10.1074/jbc.M116.738898. 10.1074/jbc.M116.738898 PMC5000057 27402852

[46] Montagna W, CHASE HB, HAMILTON JB (1951). The distribution of glycogen and lipids in human skin. J Invest Dermatol.

[47] Downie MM, Kealey T (1998). Lipogenesis in the human sebaceous gland: glycogen and glycerophosphate are substrates for the synthesis of sebum lipids. J Invest Dermatol.

[48] Figlak K, Williams G, Bertolini M, Paus R, Philpott MP (2021). Human hair follicles operate an internal Cori cycle and modulate their growth via glycogen phosphorylase. Sci Rep.

[49] Reichenbach B, Classon J, Aida T, Tanaka K, Genander M, Göritz C (2018). Glutamate transporter Slc1a3 mediates inter-niche stem cell activation during skin growth. EMBO J.

[50] Okumoto K, Tamura S, Honsho M, Fujiki Y (2020). Peroxisome: Metabolic Functions and Biogenesis. Adv Exp Med Biol.

[51] Gorgas K, Völkl A. Peroxisomes in sebaceous glands. IV. Aggregates of tubular peroxisomes in the mouse Meibomian gland. Histochem J. 1984;16:1079–98. doi:10.1007/BF01002896. 10.1007/BF01002896 6500992

[52] Zhang F, Zhang Y, Deng Z, Xu P, Zhang X, Jin T, Liu Q (2016). Genetic variants in the acylphosphatase 2 gene and the risk of breast cancer in a Han Chinese population. Oncotarget.

[53] Albrektsen T, Richter HE, Clausen JT, Fleckner J (2001). Identification of a novel integral plasma membrane protein induced during adipocyte differentiation. Biochem J.

[54] Eguchi J, Wada J, Hida K, Zhang H, Matsuoka T, Baba M (2005). Identification of adipocyte adhesion molecule (ACAM), a novel CTX gene family, implicated in adipocyte maturation and development of obesity. Biochem J.

[55] Bai N, Lu X, Jin L, Alimujiang M, Ma J, Hu F (2022). CLSTN3 gene variant associates with obesity risk and contributes to dysfunction in white adipose tissue. Mol Metab.

[56] Leonard AE, Bobik EG, Dorado J, Kroeger PE, Chuang LT, Thurmond JM (2000). Cloning of a human cDNA encoding a novel enzyme involved in the elongation of long-chain polyunsaturated fatty acids. Biochem J.

[57] Jääskeläinen T, Makkonen H, Palvimo JJ (2011). Steroid up-regulation of FKBP51 and its role in hormone signaling. Curr Opin Pharmacol.

[58] Wanders RJA, Duran M, Loupatty FJ (2012). Enzymology of the branched-chain amino acid oxidation disorders: the valine pathway. J Inherit Metab Dis.

[59] Wirnsberger G, Zwolanek F, Stadlmann J, Tortola L, Liu SW, Perlot T (2014). Jagunal homolog 1 is a critical regulator of neutrophil function in fungal host defense. Nat Genet.

[60] Lagrost L, Masson D (2022). The expanding role of lyso-phosphatidylcholine acyltransferase-3 (LPCAT3), a phospholipid remodeling enzyme, in health and disease. Curr Opin Lipidol.

[61] Chu C-H, Cheng D (2007). Expression, purification, characterization of human 3-methylcrotonyl-CoA carboxylase (MCCC). Protein Expr Purif.

[62] Chen Y-Y, Zhang X-N, Xu C-Z, Zhou D-H, Chen J, Liu Z-X (2021). MCCC2 promotes HCC development by supporting leucine oncogenic function. Cancer Cell Int.

[63] Kara M, Axton RA, Jackson M, Ghaffari S, Buerger K, Watt AJ (2015). A Role for MOSPD1 in Mesenchymal Stem Cell Proliferation and Differentiation. Stem Cells.

[64] Koch J, Pranjic K, Huber A, Ellinger A, Hartig A, Kragler F, Brocard C (2010). PEX11 family members are membrane elongation factors that coordinate peroxisome proliferation and maintenance. J Cell Sci.

[65] Kim PK, Mullen RT, Schumann U, Lippincott-Schwartz J (2006). The origin and maintenance of mammalian peroxisomes involves a de novo PEX16-dependent pathway from the ER. J Cell Biol.

[66] Gakh O, Cavadini P, Isaya G (2002). Mitochondrial processing peptidases. Biochim Biophys Acta.

[67] Shivalingappa PKM, Sharma V, Shiras A, Bapat SA (2021). RNA binding motif 47 (RBM47): emerging roles in vertebrate development, RNA editing and cancer. Mol Cell Biochem.

[68] Didier C, Broday L, Bhoumik A, Israeli S, Takahashi S, Nakayama K (2003). RNF5, a RING finger protein that regulates cell motility by targeting paxillin ubiquitination and altered localization. Mol Cell Biol.

[69] Ott C, Ross K, Straub S, Thiede B, Götz M, Goosmann C (2012). Sam50 functions in mitochondrial intermembrane space bridging and biogenesis of respiratory complexes. Mol Cell Biol.

[70] Torriglia A, Martin E, Jaadane I (2017). The hidden side of SERPINB1/Leukocyte Elastase Inhibitor. Semin Cell Dev Biol.

[71] Krycer JR, Fazakerley DJ, Cater RJ, CThomas K, Naghiloo S, Burchfield JG (2017). The amino acid transporter, SLC1A3, is plasma membrane-localised in adipocytes and its activity is insensitive to insulin. FEBS Lett.

[72] Navarro-Imaz H, Ochoa B, García-Arcos I, Martínez MJ, Chico Y, Fresnedo O, Rueda Y (2020). Molecular and cellular insights into the role of SND1 in lipid metabolism. Biochim Biophys Acta Mol Cell Biol Lipids.

[73] Hwang SO, Boswell SA, Seo J-S, Lee SW (2008). Novel oxidative stress-responsive gene ERS25 functions as a regulator of the heat-shock and cell death response. J Biol Chem.

[74] Alon A, Schmidt HR, Wood MD, Sahn JJ, Martin SF, Kruse AC (2017). Identification of the gene that codes for the σ2 receptor. Proc Natl Acad Sci U S A.

[75] Antonicka H, Choquet K, Lin Z-Y, Gingras A-C, Kleinman CL, Shoubridge EA (2017). A pseudouridine synthase module is essential for mitochondrial protein synthesis and cell viability. EMBO Rep.

