# Supplementary material for: Skin single-cell transcriptomics reveals a core of sebaceous gland-relevant genes shared by mice and humans
Source: BMC Genomics. 2024 Feb 3;25:137. doi: 10.1186/s12864-024-10008-8 (PMC10837983; doi:10.1186/s12864-024-10008-8)
Supplement: Supplementary file 1 — Additional file 1: Supplementary Table 1. Non-default oposSOM parameters applied in this work. [file 12864_2024_10008_MOESM1_ESM.docx]

**Supplementary Table 1.** Non-default oposSOM parameters applied in this work.

| **Parameter** | **Value** | **Basis** |
| --- | --- | --- |
| Training.extension | 6 (predefined IFE populations)  25 (SG-populations only) | Avoid bias by pre-definition    Lower training results in bad separated groups, higher extension generates many groups with few samples |
| dim.1stLvlSom | 30 | Previous own work |
| group.labels | Predefined cluster  Unsupervised (SG-population only) | Joost et al. 2016; Cheng et al. 2018 |
