# Supplementary material for: Skin single-cell transcriptomics reveals a core of sebaceous gland-relevant genes shared by mice and humans
Source: BMC Genomics. 2024 Feb 3;25:137. doi: 10.1186/s12864-024-10008-8 (PMC10837983; doi:10.1186/s12864-024-10008-8)
Supplement: Supplementary file 2 — Additional file 2:Supplementary Figure 1. Original mouse (left) and human (right) epidermal cell transcriptome visualized with t-distributed stochastic neighbour embedding maps and corresponding legends showing sebaceous gland cells as discrete population. Figures are reproduced from Joost et al. (2016) and Cheng et al. (2018) under the CC BY-NC-ND license (http://creativecommons.org/licenses/by-nc-nd/4.0). Supplementary Figure 2. oposSOM-generated expression portraits (left) of the subpopulations in mouse scRNA-seq samples; sebaceous samples are thereby clearly discriminated from all remaining cell populations. Within the corresponding spots of strong expressed metagenes identified by oposSOM (right), Spot K represents the SG sample exclusive spot. Supplementary Figure 3. oposSOM-generated expression portraits (left) of the subpopulations in human scRNA-seq samples; sebaceous samples are thereby clearly discriminated from all remaining cell populations. Within the corresponding spots of strong expressed metagenes identified by oposSOM (right), Spot J represents the SG sample exclusive spot. Supplementary Figure 4. oposSOM-generated expression portraits of the sebaceous gland population in human (left) and mouse (bottom) scRNA-seq samples; The group overlap (top right) does not provide a distinct mouse to human group matching. The color code corresponds to the normalized appearance of homologous genes (red indicates the highest incidence). [file 12864_2024_10008_MOESM2_ESM.pdf]

Supplementary Information for

Skin single-cell transcriptomics reveals a core of sebaceous gland-relevant genes shared by mice and humans

Torsten Thalheim[1*], Marlon R. Schneider[2✉]

[1] Interdisciplinary Institute for Bioinformatics (IZBI), University of Leipzig, Härtelstr. 16-18, 04107 Leipzig, Germany
[2] Institute of Veterinary Physiology, University of Leipzig, An den Tierkliniken 7, 04103, Leipzig, Germany

* Present Address: Deutsches Biomasseforschungszentrum gGmbH, Torgauer Str. 116, 04347 Leipzig
✉ E-mail: marlon.schneider@vetmed.uni-leipzig.de

This PDF file includes:
Supplementary Figures 1-4

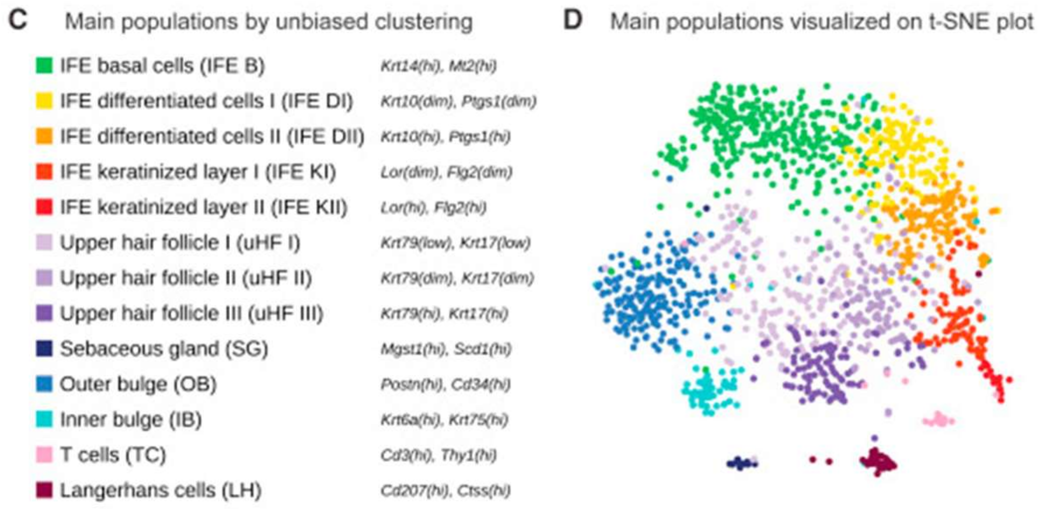

Mouse (Joost et al. 2016)

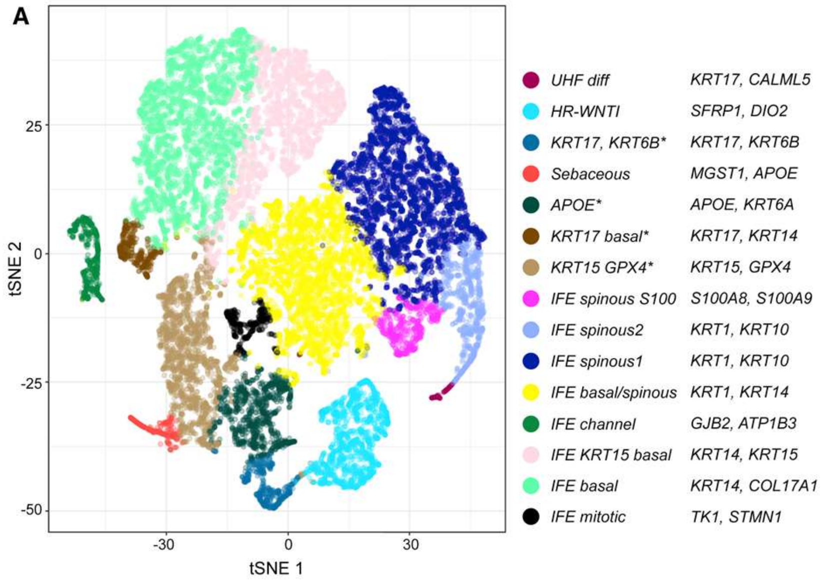

Human (Cheng et al. 2018)

Supplementary Figure 1. Original mouse (left) and human (right) epidermal cell transcriptome visualized with t-distributed stochastic neighbour embedding maps and corresponding legends showing sebaceous gland cells as discrete population. Figures are reproduced from Joost et al. (2016) and Cheng et al. (2018) under the CC BY-NC-ND license (http://creativecommons.org/licenses/by-nc-nd/4.0).

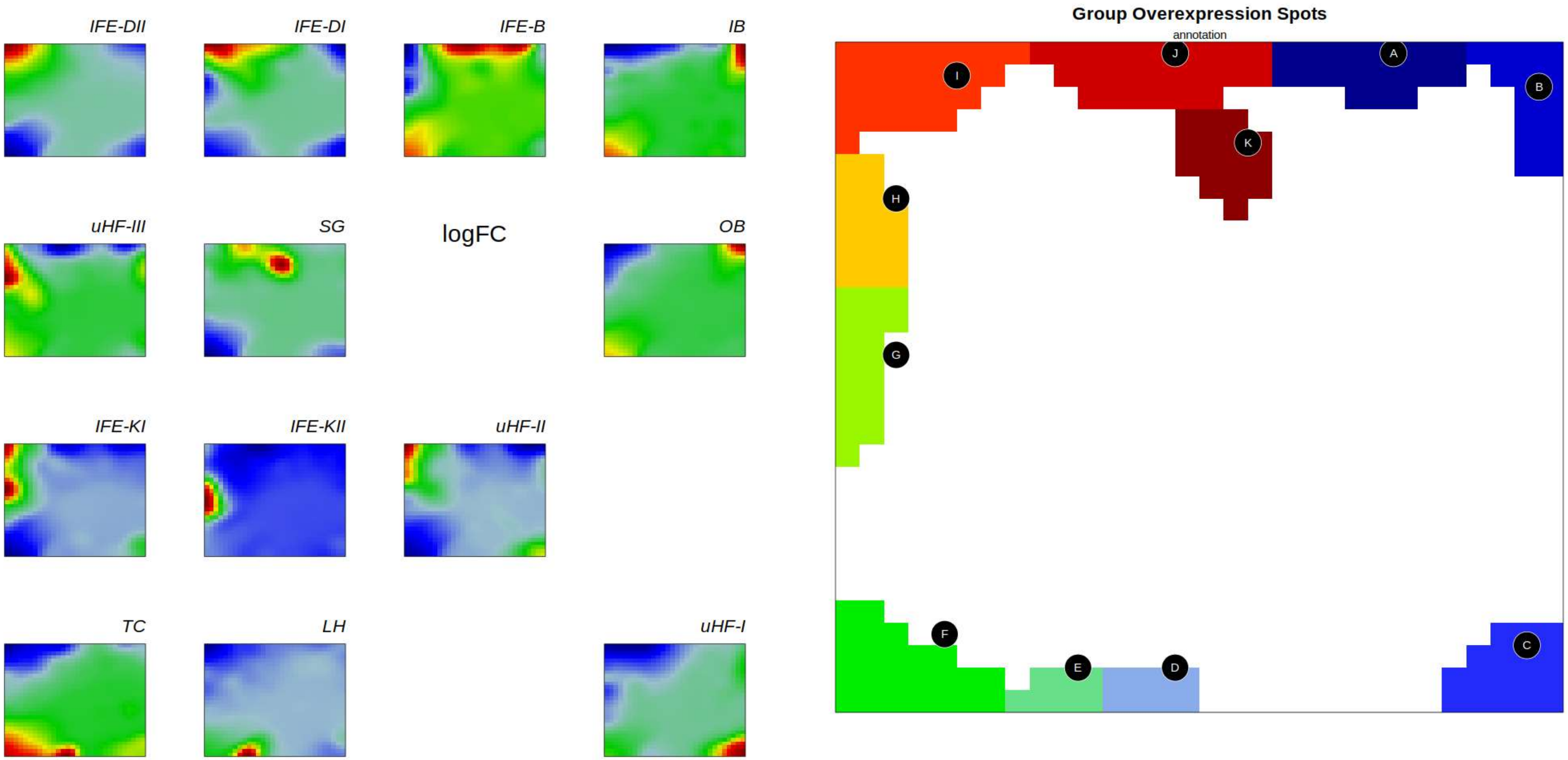

Supplementary Figure 2. oposSOM-generated expression portraits (left) of the subpopulations in mouse scRNAseq samples; sebaceous samples are thereby clearly discriminated from all remaining cell populations. Within the corresponding spots of strong expressed metagenes identified by oposSOM (right), Spot K represents the SG sample exclusive spot.

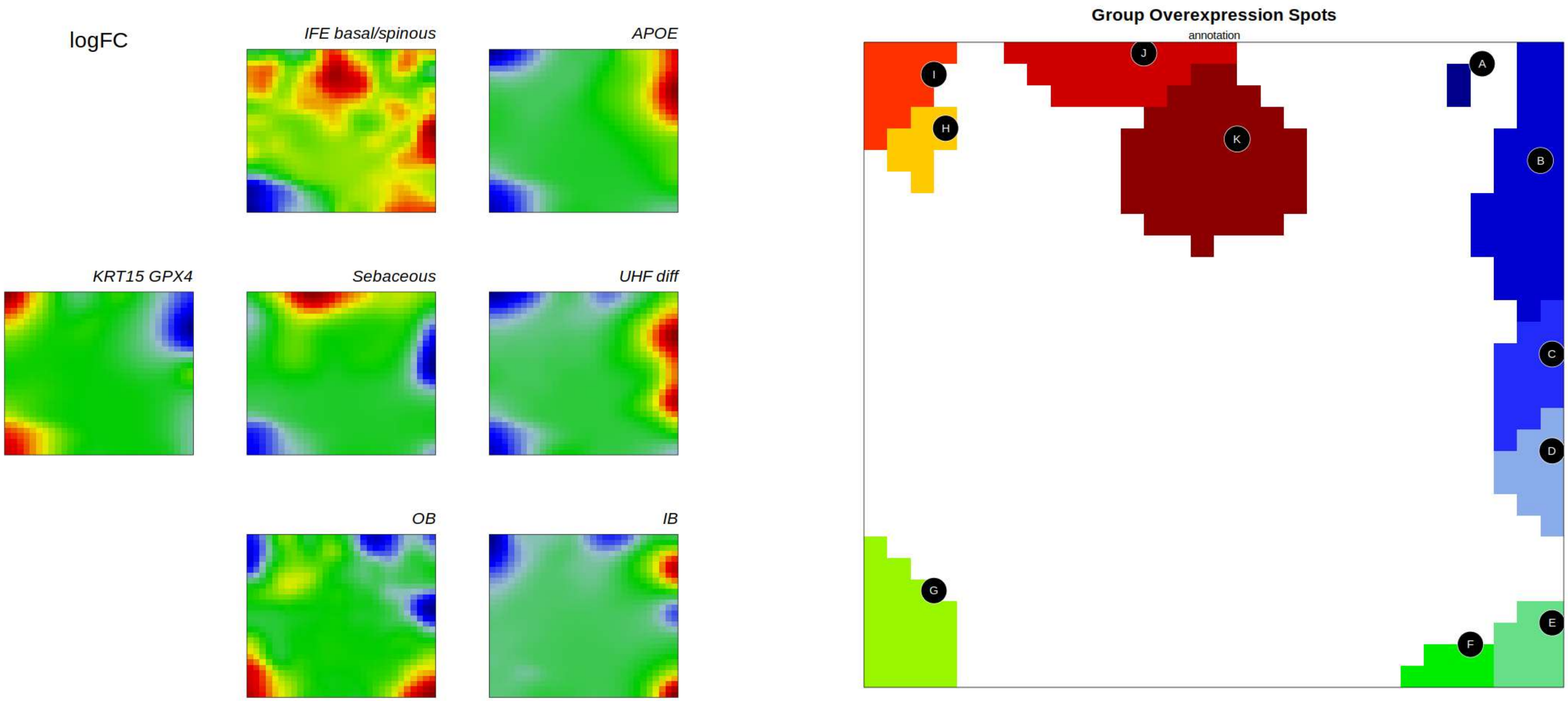

Supplementary Figure 3. oposSOM-generated expression portraits (left) of the subpopulations in human scRNAseq samples; sebaceous samples are thereby clearly discriminated from all remaining cell populations. Within the corresponding spots of strong expressed metagenes identified by oposSOM (right), Spot J represents the SG sample exclusive spot.

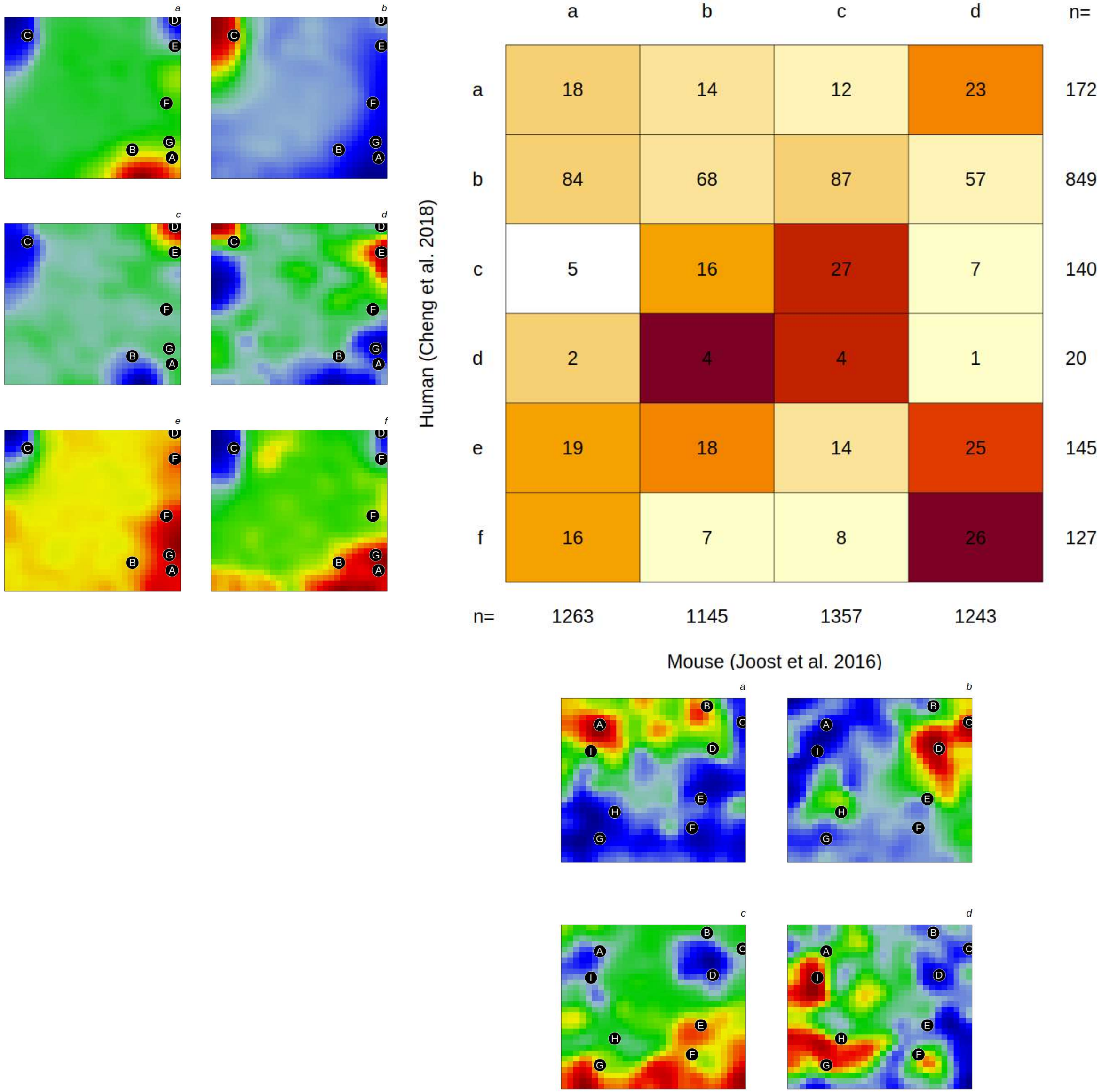

	a	b	c	d	n=
a	18	14	12	23	172
b	84	68	87	57	849
c	5	16	27	7	140
d	2	4	4	1	20
e	19	18	14	25	145
f	16	7	8	26	127
n=	1263	1145	1357	1243	

Supplementary Figure 4. oposSOM-generated expression portraits of the sebaceous gland population in human (left) and mouse (bottom) scRNAseq samples; The group overlap (top right) does not provide a distinct mouse to human group matching. The color code corresponds to the normalized appearance of homologous genes (red indicates the highest incidence).
